# Striatal and Hippocampal Involvement in Motor Sequence Chunking Depends on the Learning Strategy

**DOI:** 10.1371/journal.pone.0103885

**Published:** 2014-08-22

**Authors:** Ovidiu Lungu, Oury Monchi, Geneviève Albouy, Thomas Jubault, Emanuelle Ballarin, Yves Burnod, Julien Doyon

**Affiliations:** 1 Unité de Neuroimagerie Fonctionelle (UNF), Montréal, Canada; 2 Centre de recherche de l'Institut universitaire de gériatrie de Montréal, Montréal, Canada; 3 Département de Psychiatrie, Université de Montréal, Montréal, Canada; 4 Center for Research in Aging, Donald Berman Maimonides Geriatric Center, Montréal, Canada; 5 Département de Psychologie, Université de Montréal, Montréal, Canada; 6 Graduate Program in Neuropsychology, Université de Lyon 2, Lyon, France; 7 INSERM U678, Université de Paris VI Jussieu, Paris, France; 8 Département de Radiologie, Université de Montréal, Montréal, Canada; Harvard Medical School, United States of America

## Abstract

Motor sequences can be learned using an incremental approach by starting with a few elements and then adding more as training evolves (e.g., learning a piano piece); conversely, one can use a global approach and practice the whole sequence in every training session (e.g., shifting gears in an automobile). Yet, the neural correlates associated with such learning strategies in motor sequence learning remain largely unexplored to date. Here we used functional magnetic resonance imaging to measure the cerebral activity of individuals executing the same 8-element sequence after they completed a 4-days training regimen (2 sessions each day) following either a global or incremental strategy. A network comprised of striatal and fronto-parietal regions was engaged significantly regardless of the learning strategy, whereas the global training regimen led to additional cerebellar and temporal lobe recruitment. Analysis of chunking/grouping of sequence elements revealed a common prefrontal network in both conditions during the chunk initiation phase, whereas execution of chunk cores led to higher mediotemporal activity (involving the hippocampus) after global than incremental training. The novelty of our results relate to the recruitment of mediotemporal regions conditional of the learning strategy. Thus, the present findings may have clinical implications suggesting that the ability of patients with lesions to the medial temporal lobe to learn and consolidate new motor sequences may benefit from using an incremental strategy.

## Introduction

Motor sequences are ubiquitous in everyday life, from simple behaviors such as preparing a cup of coffee to complex activities like speaking and dancing. As such, numerous studies in the past have investigated the neuronal correlates and mechanisms implicated in motor sequence acquisition [1], [2]. As part of procedural memory processes [3], motor sequence learning is thought to take place in stages [4], whereby considerable improvements in motor performance are known to occur rapidly during the early stage of the acquisition process. These improvements are then followed by smaller gains spread out over longer periods of time across subsequent practice sessions in later stages. A large body of neuroimaging evidence has revealed that motor sequence learning is mediated by the cortico-striatal and cortico-cerebellar circuits in the early stage, regardless of whether the subject knows the sequence explicitly or not before practice begins, and by the cortico-striatal system during the later learning phases [2], [5]. Yet changes in hippocampal activity has also been reported to be associated with both implicit and explicit motor sequence learning [6]–[9], hence highlighting its ability to associate discontinuous but structured information.

Recently, there has been an increased interest in investigating the behavioral and neurophysiological determinants of long-term motor sequence memory consolidation. This process can be facilitated by sleep (both day and night sleep) and it was found to be dependent upon the cognitive functions recruited during the acquisition process [10]–[14]. Moreover, consolidation appears to be based on increased activity within the striatum [15], and mediotemporal lobe (MTL), especially the hippocampus [6], [8], [13], [17]–[19]. Interestingly, the interaction of both striatum and hippocampus with frontal areas during initial training appears to facilitate the implementation of reproducible motor behavior [7]. While the role of the cortico-striatal circuit in long-term motor sequence acquisition seems to be in the grouping of sequence elements into single-action units (i.e., in creating chunks) [20]–[24], the functional contribution of the hippocampus could be in the detection and formation of higher-order sequential associations [9], [25]–[27] and the stabilization of the motor performance [7]. Yet one important issue that has entirely been overlooked so far by the neuroimaging literature is the extent to which the involvement of neural substrates found in later stages of motor sequence learning, such as the striatum and hippocampus, is modulated by the *type* of training regimens or learning strategies employed for acquiring a new motor skilled behavior.

In everyday life, when learning explicit sequences, such as steps of a dance or to play a new piece of music, we usually use an incremental approach, that is we start by practicing a part of the sequence of movements to be learned, and then expand to include more and more elements until we are able to perform it wholly. Yet in other occasions (e.g., learning to shift gears to manually drive an automobile), we may need to practice the whole sequence all at once (global approach) before we put it into use (e.g., driving in traffic). When using the incremental training regimen, the sequence representation changes and gradually builds up in complexity, whenever new elements are added to the sequence. This gradual build-up of sequence representation should be associated with a slowdown of motor performance during learning. For instance, in a series of behavioral experiments, Ganor-Stern and colleagues [28] showed that changes in earlier versus later sequence elements (i.e. the 3^rd^ versus the 6^th^ element of an 7-element sequence) were found to lead to a greater impairment in performance. These findings suggest, on the one hand, that participants built-up the sequence representation by chunking together the earlier elements first, then followed by the later elements, but also showed that motor performance is impaired whenever sequence representation is changed. By contrast, with the global learning strategy, stable and complex sequence representations are thought to be formed. For instance, it was shown that abstract, effector independent sequence representations can form rapidly and early on during the motor skill acquisition process, especially when the sequence is known explicitly [29], [30], while motor, effector dependent representations are formed slowly after extended periods of training [29].

Given the scarcity of studies comparing directly these two learning strategies, some important knowledge gaps remain to be addressed. For instance, to what extent differences in sequence representation, as a result of incremental versus global training, are manifested behaviorally, and more specifically in the way sequence elements are chunked or grouped together? Also, neuroimaging studies on long-term explicit motor sequence learning employed exclusively a global learning strategy, whereby the same sequence was practiced over many training sessions, spanning days or weeks. In most cases, the contribution of striatum and hippocampus in this process has been assessed by comparing motor performance and brain activity between sessions (i.e. last vs. first). Thus,, it is still unknown whether the long-term maintenance of the skill (i.e. producing the well-rehearsed and consolidated sequence at the end of multi-session training) is subserved by different neural substrates as a result of employing different learning strategies (i.e. incremental versus global). Here we directly compare the production of the same explicit motor sequence and we investigate, using fMRI, the contribution of the cortico-striatal and MTL regions in this process at the end of a 4-day incremental versus global behavioral training regimen of a sequence of movements.

## Materials and Methods

### Participants

Thirty-two subjects aged 19–36 years old were initially recruited for this study. About two thirds of the participants were female (23 out of 32) and the participants' recruitment was done via advertisement on the university campus. None of the subjects had a history of neurological or psychiatric disorders as revealed by anamnesis prior to the experiment. Out of the 32 participants, 4 did not complete the behavioral training regimen and consequently, they were excluded from the data analysis. Six others did terminate the behavioral training, but did not participate in the imaging session due to unanticipated technical problems with the scanner's cooling system during the week they were trained; and thus their data were also excluded from the analysis. The data from the remaining 22 subjects is reported here.

### Ethics statement

All participants gave their voluntary consent and signed a form, which was approved by the joint research ethics committee of the Regroupement Neuroimagerie Québec at the “Centre de recherche, Institut universitaire de gériatrie de Montréal”. This committee, which approved the study and its experimental procedures, follows the guidelines of the Tri-Council Policy Statement of Canada, the civil code of Quebec, the Declaration of Helsinki and the code of Nuremberg.

### Experimental design and procedure

**Figure 1:**
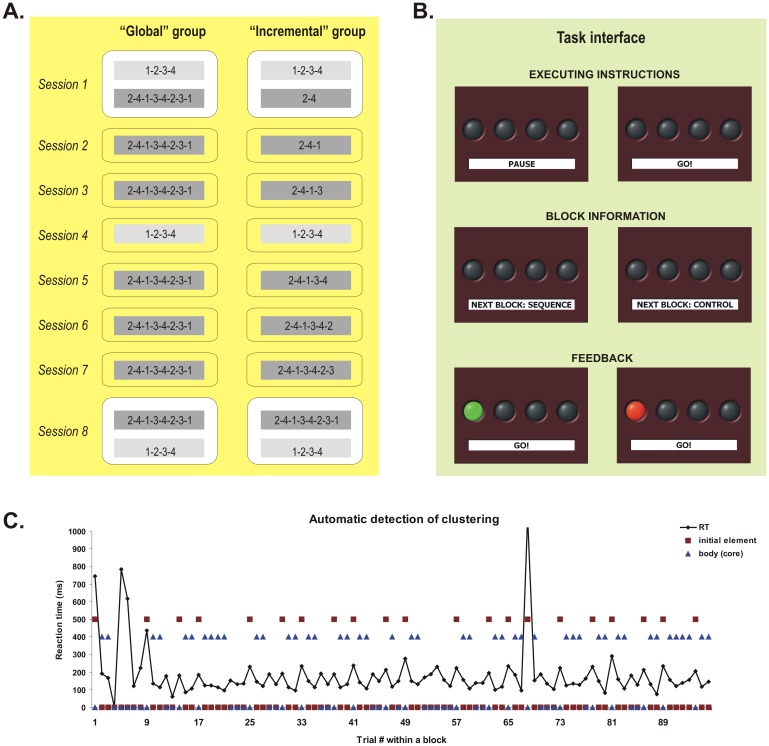
Experimental groups and sessions, the task and the automatic chunking procedure. **A.** Control (light gray) and target (dark gray) sequences practiced by the two groups of subjects across training sessions. The numbers 1 to 4 correspond to four fingers of the left hand, from the little (1) to the index (4) finger. **B.** The task interface presenting the executing instructions, the block information and the type of feedback after each trial. **C.** Reaction time (RT) in a block for a given subject and the classification of trials into initial element of a chunk (red squares) and the body or core of the chunk (blue triangles) by our automatic clustering algorithm.

All participants followed a four-day behavioral training regimen with the aim of learning an explicit 8-element motor sequence by the end of it. In the fifth day, learning was assessed in a test session performed in the MRI scanner. Thus, the training routine always started on Monday and finished on Thursday, with two daily sessions (morning and afternoon) of behavioral training. The following day in the morning (always on Friday), subjects were tested on the motor sequence learning task while their brain activity was recorded. One group, ‘Global’ (n_1_ = 11), always practiced the entire 8-element sequence in each of the training sessions, except the 4^th^ (Global training). By contrast, the other group, ‘Incremental’ (n_2_ = 11), started off with only two elements in the first training session, and was then asked to practice an increasingly more difficult sequence as one more element was added to the sequence on each subsequent session (except the 4^th^) (Incremental training) (see Figure 1A). Also, during behavioral training, each group was exposed to two conditions: “Sequence” where subjects practiced the 8-element target sequence, either globally or incrementally, and “Control” where participants practiced a simpler, 4-element sequence (for details, see task description). In all but the first and last training sessions of the Sequence condition subjects executed 16 blocks of 96 sequential movements each, interspersed with 10 seconds rest periods. In the first and last sessions subjects also performed separately 16 blocks with 96 trials each in the Sequence condition and 16 blocks with the same number of trials in the Control condition. In the 4^th^ session, participants were exposed only to the Control condition (Figure 1A), again for 16 blocks with 96 trials. The next morning, after the 4-day behavioral training, subjects were tested in a single session with 5 imaging runs of 8 blocks each, interspersed with 30 seconds rest periods. In each scanning run, they were exposed to alternating pairs of blocks within the Sequence or Control condition. This particular distribution of the Sequence and Control training sessions over the 4-day training schedule was chosen to ensure that subjects in the Incremental group trained on a different sequence in each session. In addition, the decision to have morning and afternoon training sessions, rather than one per day or at bigger intervals, was made to minimize subject attrition rate because, otherwise, the training would have required subjects to commit to the study for more than a week.

### Task description

Subjects were tested using a version of the explicit motor sequence learning task similar to that developed by Karni and colleagues [4], [31], [32]. In this task, participants were required to perform self-generated finger movements with their non-dominant (left) hand. They were shown four grey discs arranged in a row on a computer screen, either directly (during behavioral training) or through a mirror embedded within the head coil (in the scanner, at retest). Each disk corresponded to one of four buttons on a numerical keyboard (behavioral training) or on a MR compatible button box (in the scanner). Under the row of discs, subjects were also shown a small window comprising the following written instruction cues: “Pause” when subjects had to rest between blocks, or “Go”, when they had to execute the finger movements (Figure 1B). Before the onset of each session, subjects were explicitly informed about the sequence of movements that had to be executed in each block. In the Sequence condition, participants in the Global group were asked to practice the following finger sequence: 2-4-1-3-4-2-3-1, where 1 represents the small finger and 4 represents the index finger of the non-dominant hand. By contrast, participants in the Incremental group were required to practice only parts of this sequence, with one additional element being added to the sequence on each subsequent session. For instance, in the first session they were asked to perform the first two elements of the sequence (2-4) only; in the second session – the first three elements (2-4-1), and so on, until the last training session where subjects had to perform all 8 elements of the sequence. This sequence was chosen for the following reasons: (1) all elements appear with the same frequency, (2) it is complex in structure because there are only second-order conditional transitions between elements (i.e. one needs to know a minimum of two immediately preceding elements to predict the current element), and (3) it needs some amount of practice in order to achieve asymptotic performance because there are no easy consecutive triplets (e.g. 1-2-3 or 4-3-2), which can be performed automatically. A simple sequence (1-2-3-4) was also employed as a control condition, which was administered during the 1^st^, 4^th^ and 8^th^ practice sessions (see Figure 1A). This sequence was chosen as control because it can automatize rapidly (i.e. asymptotic performance) due to the presence of consecutive elements and single-order conditional transitions. Subjects started each session with a 10 seconds rest period (30 seconds during the imaging session), indicated by the instruction cue. Five seconds prior to each block, the instruction cue informed the subject about the type of movements to be executed next: “Sequence”, for the target sequence or “Control” for the control condition (Figure 1B). Each block of practice was initiated as soon as the cue changed to “Go”, hence instructing the subjects that they had to start executing the sequence of movements at will. They were instructed to execute the movements as quickly as possible, while making as few errors as possible. Immediately after the subject pressed a button, the corresponding discs on the screen were switched to green (if subjects chose the correct button, a hit) or to red (if subjects chose a different button, an error). The location of the illuminated disc always indicated the correct location for that particular trial (Figure 1B). If an error was committed, subjects were instructed to continue the sequence in the proper order, with the next element. Therefore, the task design provided subjects in each trial with a visual feedback regarding their own performance.

### Behavioral data analysis

#### Reaction time

Several dependent measures have been computed in past studies using serial reaction time task paradigms. For instance, in the classical serial reaction time task [3], learning-specific effects have been assessed by comparing the difference in reaction time between sequential and randomly presented elements. In our task, because motor responses were self-generated, it was impossible to have a random condition. Therefore, different learning metrics were employed. One was to compare the improvements in sequential motor performance time across sessions. Yet, by design, this was only possible in the Global group where the sequence was unchanged from one session to the next. Alternatively, the performance in the Control condition (the sequence 1-2-3-4) can be taken as a benchmark for automaticity or asymptotic sequential performance; thus, the smaller the difference in performance between Sequence and Control conditions, the better the learning. By design, the two groups, Global and Incremental, did not practice the same number of elements from the target sequence during the behavioral training period (i.e. the Global group practiced always all 8 elements, whereas the Incremental group started with 2 elements in the first session and arrived to practice the whole 8-element sequence only in the last training session). Thus, the behavioral data could only be analyzed in situations where a match between the two groups was found in this regard. To this end, we analyzed the blocks from the Control condition during Sessions 1 and 4 and both the blocks from the Control and Sequence condition in the last behavioral training session (Session 8) and at test (fMRI session). For this analysis, we employed a general linear model (GLM) approach, where the Group (Global vs. Incremental), Session (Sessions 1^st^ vs 4^th^ vs 8^th^ for Control condition only) or Group and Condition (Control vs. Sequence, during the 8^th^ training or fMRI sessions) were used as fixed independent factors, and the reaction time (RT) of correct trials was the dependent measure. We used RTs per trial rather than the correct number of sequences per block or the average time to execute a sequence as a dependent variable, because we were also interested in obtaining a measure of the subject's ability to chunk sequence elements together based on differences in RTs from one trial to the next (see the “Trial chunking” section below). Subjects were always entered as a random factor in each analysis. Sidak tests were employed to test post-hoc differences between means in order to account for the number of multiple pairwise comparisons.

#### Error rates

In most serial reaction time paradigms, error rates (i.e. proportion of errors over the total number of trials) are usually low and rarely constitute the main variable of interest. Nevertheless, we measured, reported and analyzed them in the current study. We computed the error rate for each block of 96 trials, in each condition, session and for each participant. Then, these block-related error rates were averaged by session, condition and group. We then used GLM models to assess differences in error rates as a function of session, condition and group both for the behavioral training data, as well as for the data acquired during the imaging session.

#### Trial chunking

A separate analysis was performed on the chunking of movements within a given block; the number and length of these chunks provide insights into the learning strategy that subjects used based upon the type of training (Global vs. Incremental) and allows for group comparison with respect to this aspect. As shown in previous studies [20], [22], [33]–[35] people tend to group the elements of a motor sequence in chunks or clusters, like dialing a long phone number by chunking its different parts such as the country code, area code, and number, for example. These chunks are characterized by a fast and similar reaction time for each of its elements, except the first. For example, previous investigations have shown that, during motor chunking, the subject's reaction time to execute the first sequence element is always slower due to transitions between chunks (i.e. the end of one and beginning of another), probably reflecting a starting cost or a higher memory load due to the fact that the previous cluster is discarded and another one is loaded [20], [36], [37]. In order to analyze the difference in chunking strategy between the two Global and Incremental groups, we devised an automatic algorithm that can categorize a given trial in a block as being the first element of a chunk [INITIAL], part of the rest of the elements in that chunk [BODY], or as being in neither one category or the other [OTHER]. This algorithm first computed the mean RT and the standard error of the mean for each block of trials and for each subject. Then, all correct first trials in each block and all the correct trials immediately after an error were automatically classified as [INITIAL]. If the differences in reaction time between the (***i***)^th^ correct trial and the two adjacent correct trials, (***i−1***)^th^ and (***i+1***)^th^, were *both* higher than three standard errors of the mean for that particular block AND higher than two standard errors of the mean when compared with the (***i+2***)^th^ correct trial, then the (***i***)^th^ trial was categorized as INITIAL. Correct trials following an INITIAL trial were categorized as part of the BODY as long as the difference in reaction time between them remained equal or less than two standard errors of the mean. The errors and the rest of the correct trials that could not be classified as [INITIAL] or [BODY] were categorized as OTHER. Panel C in Figure 1 shows the reaction time in a block of trials for one participant, as well as the trial's categorization in either an [INITIAL] or [BODY] element, It is interesting to note that the first element of the sequence (trial #1, #9, #17, etc.) was overwhelmingly categorized as an [INITIAL] element by this automatic algorithm.

#### Variability in performance

Another way to measure the effects of our experimental manipulation on learning the motor sequence is to analyze the subjects' variability in movement execution. The more the sequence becomes consolidated and the more subjects are able to reproduce it in the same fashion, the lower the variability [38]. The evolution in performance variability is believed to reflect the implementation of a performance mode that would represent the whole sequence of movements in motor memory [38]. In order to perform this analysis for the behavioral training sessions, we computed the standard deviation for the RTs of correct trials only for each Sequence block, in each session and for each subject. We averaged the data across blocks for each subject in each session, hence obtaining a single value per subject and per session. These standard deviation measures were then used as dependent variable in a mixed repeated measures analysis of variance (ANOVA), with sessions as a within-subject factor and groups (Global vs. Incremental) as a between-subjects factor. A similar analysis approach was used for the behavioral data acquired during the fMRI session, except that instead of averaging the measures for the entire session, we averaged the standard deviations of RTs across the blocks of a given fMRI run. Therefore, in the mixed repeated-measures ANOVA, we used the run as a within-subject factor, while preserving the group as between-subjects factor.

### Imaging parameters

A 3 Tesla whole Body MR System (Magnetom TIM, Siemens Medical Systems, and Erlangen, Germany) was used for image acquisition. Prior to the functional run, 176 structural images were acquired in sagittal plane by using an MPRAGE imaging sequence (TR = 13 ms; TE = 4.92 ms; FA = 25°; FoV = 256 mm^2^; matrix size = 256×256; slice thickness = 1 mm, voxel size = 1 mm^3^). Then, whole brain fMRI was performed using an echo-planar imaging (EPI) sequence measuring blood oxygenation level dependent (BOLD) signal (TR = 2510 ms; TE = 30 ms; FA = 90°; FoV = 220 mm^2^; matrix size = 64×64; slice thickness = 3.4 mm, voxel size = 3.4 mm^3^; 41 slices). The functional slices covered the whole brain, were oriented in transverse plane and were angled to be parallel to the AC-PC line. An inline retrospective motion correction algorithm was employed while the EPI images were acquired. A total of 205 functional volumes were recorded in each functional run.

### Preprocessing of fMRI data

Brain Voyager QX (Brain Innovation B.V., Maastricht, the Netherlands) software was used for fMRI data preprocessing and analysis. The functional bi-dimensional images of every subject were preprocessed to correct for the difference in time slice acquisition (slice scan time correction). In addition to linear detrending, a high-pass filter of three cycles per time course (frequency domain) was applied to the corrected 2D slices. The functional series were then preprocessed to correct for possible motion artifacts in 3D space, and to ensure that movements in any plane did not exceed 3 mm. These functional images were subsequently used to reconstruct the 3D functional volume for every subject, and for every run. The 3D functional volumes were then aligned with the corresponding 3D anatomical volume, and both were normalized to standard Talairach space (Talairach and Tournoux, 1988. Spatial smoothing using a Gaussian kernel at 8 mm full width at half maximum (FWHM) was applied to the 3D functional data.

### Imaging data analysis

Both block-design and event-related analysis approaches were employed.

#### Block design analysis

In each group and within each run, two predictors describing the type of block or experimental conditions were defined (i.e. Sequence vs. Control). Then, three statistical contrasts were tested: one to assess group differences (Global vs. Incremental) regardless of experimental condition, one to evaluate the main effect of condition (Sequence vs. Control), regardless of the group membership and one contrast assessing the Group*Condition interaction.

#### Event-related design analysis

For the event-related design, the following predictors were defined, based on the analysis of behavioral data at individual trial level (see the clustering of trials in the behavioral data analysis): (1) first element of a chunk in the Sequence blocks [*initial SEQ*], (2) the rest of the elements of a chunk in the Sequence blocks [*body SEQ*], (3) first element of the chunk in the Control blocks [*initial CTR*], (4) the rest of the elements within a chunk in the Control blocks [*body CTR*] and (5) all other elements that could not be categorized by the algorithm in one the previous four categories [*OTHER*]. The contrasts of interest in this analysis were *initial SEQ*>*body SEQ* and *initial SEQ*<*body SEQ*, in order to identify the brain regions in which the activity was higher during chunk initiation than during the execution of main chunk core or vice versa. These contrasts were tested (1) combining both groups together, (2) as a group difference (interaction contrast) and (3) separately within each group.

In both block and event-related analyses, the predictors were first entered as fixed factors in single subject GLM's, and then the parameters of this model were subsequently entered into a second level of analysis corresponding to a random-effect GLM model that was used for group analysis [39]. The statistical parameters of this latter model were estimated voxelwise for the entire brain and activation maps were computed for various contrasts between the predictors. While these contrasts were computed and will be reported for the whole brain, our discussion of the imaging results section will focus mostly on the cortico-striatal network and mediotemporal areas known to be involved in motor sequence learning. When displaying activation maps reflecting contrasts between predictors for all of the subjects, regardless of the group membership, the false discovery rate [q(FDR)<0.05] was employed as a criterion to correct for multiple comparisons with a minimum cluster size of 108 adjacent significant isovoxels (108 cubic millimeters in volume) that surpassed this threshold. When displaying the activation maps for the contrasts reflecting group differences or performed separately for each group of subjects, we employed the same cluster size and a statistical threshold for each voxel in the cluster of at least *p<0.005* (uncorrected) for group differences contrasts and of *p<0.001* (uncorrected) for contrasts performed within each group.

Further GLM analyses were performed within the cortico-striatal and mediotemporal regions of interest that were activated significantly in previous contrasts. In these analyses the average percentage of BOLD signal change in the whole cluster was considered as dependent measure, with the group and block type as independent factors. The data for this analysis was extracted from Brain Voyager and the GLMs were analyzed by using SPSS software (SPSS Inc., Chicago, IL, USA).

## Results

### Behavioral results: Error rates and reaction time

**Figure 2:**
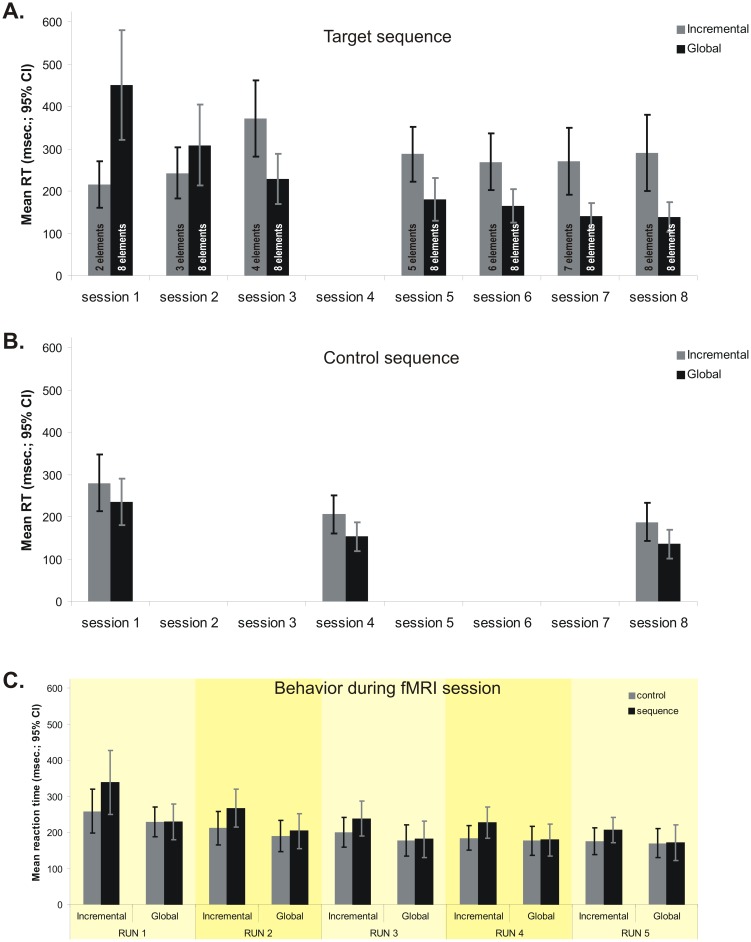
Behavioral results. **A**. Mean reaction time for the target sequence in the training sessions, for each group. **B.** Mean reaction time for the control sequence in sessions 1, 4 and 8, for each group. **C.** Mean reaction time for target and control sequences and for each group during the fMRI (9th) session.

Error rates were not significantly different as a function of any of the independent variables (session, type of block, groups), either alone or in interaction with each other (Table 1). Analysis of the mean reaction time data during correct trials in the 8^th^ session (the last behavioral practice session, where both group performed the same 8-element target sequence) as a function of experimental group (Global vs. Incremental) and the type of block (Control vs. Sequence) revealed a significant interaction effect [*F_1,20_ = 15.13; p<0.05*], as well as significant main effects of group [*F_1,20_ = 7.12; p<0.05*] and block type [*F_1,20_ = 16.48; p<0.05*]. Pairwise comparisons revealed that the interaction was driven by a significant difference between control and sequence blocks among subjects in the Incremental group (187.23 msec. vs. 290.53 msec.), whereas those in the Global group displayed similar RTs (136.27 msec. vs. 138.48 msec.) in both types of blocks (Figure 2A). These results show that, at the end of behavioral training, the Global condition group developed some level of automaticity regarding the target sequence as indicated by the fact that their reaction time was similar to that of the control sequence. Also, the main effect of group suggests that this level of automaticity in performance among participants in the Incremental group was lower than that among participants in the Global condition. However, our results suggest that the Incremental group also learned the target sequence. This is illustrated by the fact that their performance in the 8^th^ session, that is when they were required to execute the full sequence for the first time, was significantly better than that of the Global group in the first training session [*F_1,20_ = 4.34; p<0.05*]. In fact, performance of the Incremental group executing the whole 8-element sequence in the 8^th^ training session did not differ from that of the Global group in their 2^nd^ [*F_1,19_ = 0.77; p = 0.78*] session.

**Table 1:** Error rates (percentages) across sessions, type of block, experimental condition and Group.

	GROUP			
	Incremental		Global	
	*Mean*	*SEM*	*Mean*	*SEM*
**Behavioral training**				
***Control condition***				
*Session 1*	3.56	0.91	3.27	0.97
*Session 4*	3.37	0.65	1.88	0.47
*Session 8*	4.78	1.22	6.60	4.26
***Sequence condition***				
*Session 1*	4.34	0.84	6.96	1.92
*Session 2*	4.02	1.10	5.00	1.44
*Session 3*	4.04	0.79	3.69	1.35
*Session 5*	3.71	0.59	2.37	0.46
*Session 6*	5.99	1.17	2.56	0.94
*Session 7*	6.01	1.59	2.23	0.57
*Session 8*	5.37	1.26	6.26	4.06
**Test (** ***fMRI session*** **)**				
***Control condition***	3.60	0.74	1.70	0.30
***Sequence condition***	3.21	0.43	2.05	0.33

**Control condition during behavioral training.**

Session main effect: *F_2,40_ = 1.33; p = 0.27*.

Group main effect: *F_1,20_ = 0.95; p = 0.76*.

Session *Group interaction: *F_2,40_ = 0.59; p = 0.55*.

**Sequence condition during behavioral training.**

Session main effect: *F_6,120_ = 1.11; p = 0.35*.

Group main effect: *F_1,20_ = 0.22; p = 0.63*.

Session *Group interaction: *F_6,120_ = 1.63; p = 0.14*.

**Control and Sequence condition during test (fMRI session).**

Condition main effect: *F_1,20_ = 0.005; p = 0.94*.

Group main effect: *F_1,20_ = 3.63; p = 0.07*.

Condition*Group interaction: *F_1,20_ = 1.21; p = 0.28*.

RT analysis of the control sequence during the 1^st^, 4^th^ and 8^th^ training sessions revealed no significant group x session interaction [*F_2,40_ = 0.09; p = 0.91*], nor any significant main effect of group [*F_1,20_ = 2.33; p = 0.14*]. Yet the results showed a significant main effect of session [*F_2,40_ = 50.85; p<0.05*] (Figure 2B). Post-hoc Sidak tests also revealed that the two groups did not differ in their reaction time during the execution of control sequence in any of the three sessions. The latter results suggest that while the two groups improved over time for the control session, indicating a practice effect, the rate of improvement for the control sequence was similar in each group.

The RT analysis during the imaging session (9^th^) showed a significant interaction effect [*F_1,20_ = 6.35; p<0.05*] between the group and block type in the first run only (Figure 2C). For the remaining 4 runs of the sequence and control blocks, there was no significant effect of group [*F_1,20_ = 0.56; p = 0.81* for control blocks and *F_1,20_ = 0.99; p = 0.32* for sequence blocks], nor any group * run interaction [*F_3,60_ = 0.91; p = 0.44* for control blocks and *F_3,60_ = 0.84; p = 0.47* for sequence blocks]. In addition, no significant interaction was found between run and type of block (sequence vs. control), when considering the data from the Incremental [*F_3,30_ = 1.34; p = 0.27*] and Global [*F_3,30_ = 1.93; p = 0.14*] group separately. The overall 3-way interaction between group, type of block and run was also not significant [*F_3,60_ = 0.31; p = 0.81*]. Altogether, these results suggest that performance of the control and target sequences of the two groups in the last 4 fMRI runs was similar, and we thus subsequently decided to exclude the first run from the analysis of the imaging data.

### Behavioral results: clustering and variability

**Figure 3:**
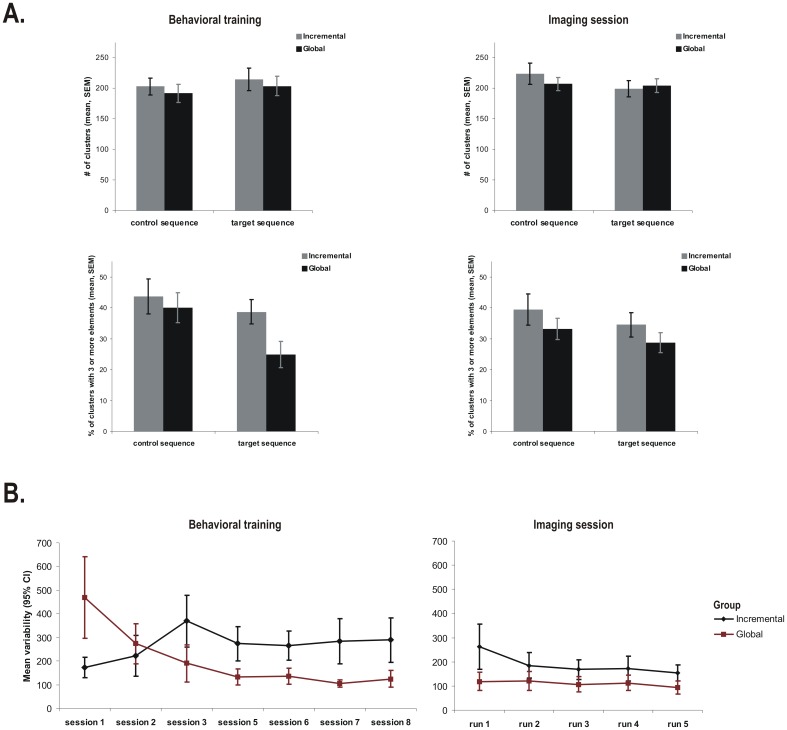
Sequence chunking and variability of performance. **A.** Mean number of clusters (first row) and the percentage of clusters with 3 or more elements (second row) in session 8th (left, behavioral) and 9th (right, imaging), for participants in the two groups. **B.** The mean variability in motor performance during the execution of the target sequence during 8 behavioral training sessions (left) and neuroimaging runs, during 9th session (right).

The clustering analysis of the 8^th^ training session (Figure 3A, left panels) showed that the number of clusters within a block detected by our automatic algorithm was not significantly affected by the Global vs. Incremental group conditions [*F_1,20_ = 0.38; p = 0.54*], the type of block [*F_1,20_ = 0.76; p = 0.39*], and the group x type of block interaction was also not significant [*F_1,20_ = 0.0004; p = 0.98*]. However, the percentage of clusters with more than 3 or 4 elements in length were both significantly higher among subjects in the Incremental than in the Global group [*F_1,20_ = 9.47; p<0.05* for the percentage of clusters with more than 3 elements and *F_1,20_ = 7.37; p<0.05* for the percentage of clusters with more than 4 elements in length]. Such findings suggest that the type of learning (global versus incremental) led, at the end of training, to the development of longer clusters among participants in the Incremental than in the Global group condition, despite the fact that the total number of clusters was comparable among the two groups.

During the last 4 runs of the imaging session, an analysis measuring trial clustering (number of clusters per block, and the percentage of clusters with more than 3 or 4 elements) revealed no significant difference between the two groups, across runs and as a function of block type [*F_3,60_ = 0.31; p = 0.81* for the total number of clusters; *F_3,60_ = 0.68; p = 0.56* for the percentage of clusters with more than 3 elements and *F_3,60_ = 1.53; p = 0.21* for the percentage of clusters with more than 4 elements]. These results (Figure 3A, right panels) indicate that the two groups chunked the sequence elements to the same degree and in similar sizes during the imaging session.

Subjects' average performance variability within blocks during the different behavioral training sessions revealed a crossover pattern (Figure 3B, left panel), where the variability decreased across sessions in the Sequence blocks for subjects in the Global group, but increased for those in the Incremental group. This pattern was expressed in a significant interaction effect between the type of group and session [*F_6,120_ = 20.54; p<0.05*]. Pairwise comparisons showed that the two groups were different in variability in all sessions (all Sidak tests p<0.05), except in the second one, where the crossover actually occurs. Moreover, after the third session, the variability in performance remained at the same level in both groups (all Sidak tests p>0.05). These results suggest that participants in the Global group became stable across sessions and less variable within session in their performance of the motor sequence during last 4 sessions, whereas those in the Incremental group were more variable within session, albeit pretty stable across sessions. In the fMRI session, the same analysis revealed again a significant interaction between the type of group and run [*F_4,80_ = 2.98; p<0.05*]. However, Sidak tests revealed that this effect came from a significant difference between the first run and the rest of them for the subjects in the Incremental group only (Figure 3B, right panel), whereas individuals in the Global group showed no change in variability across runs.

Taken together, the behavioral results suggest that despite differences observed at the end of training, the participants in the two experimental groups displayed similar motor performance, both in terms of the speed of execution and clustering during the last four runs of the imaging session. The only difference between participants in the two groups was observed at the level of motor sequence reproducibility, as measured by the level of variability level, which was always higher in the Global than in the Incremental condition, a carryover effect of the nature of training during procedural learning.

### Imaging results: block contrasts analysis

As mentioned above, data from the first run of the imaging session were excluded from the analysis because behavioral performance of the two groups differed in terms of reaction time (Fig. 2C), and because the participants in the Incremental group had a higher variability relative to the subsequent runs (Fig. 3B, right panel). This ensured that differences in speed of movement execution would not ‘contaminate’ the observed differences in brain activation, and that only the carryover effect of different training regimens would be expressed in the neuroimaging data. Yet another reason to exclude the first run stemmed from the habituation effects to the new environment. During the behavioral training sessions, the participants were seated in front of a computer screen and were asked to use a keyboard to respond. By contrast, during the fMRI session, subjects were required to lie down in the scanner in a supine position and to use a MR compatible keypad. Thus, we believe that whatever changes in the behavioral performance during the first run of the fMRI session, this could be, at least in part, attributed to changes in the body position and response device.

**Figure 4:**
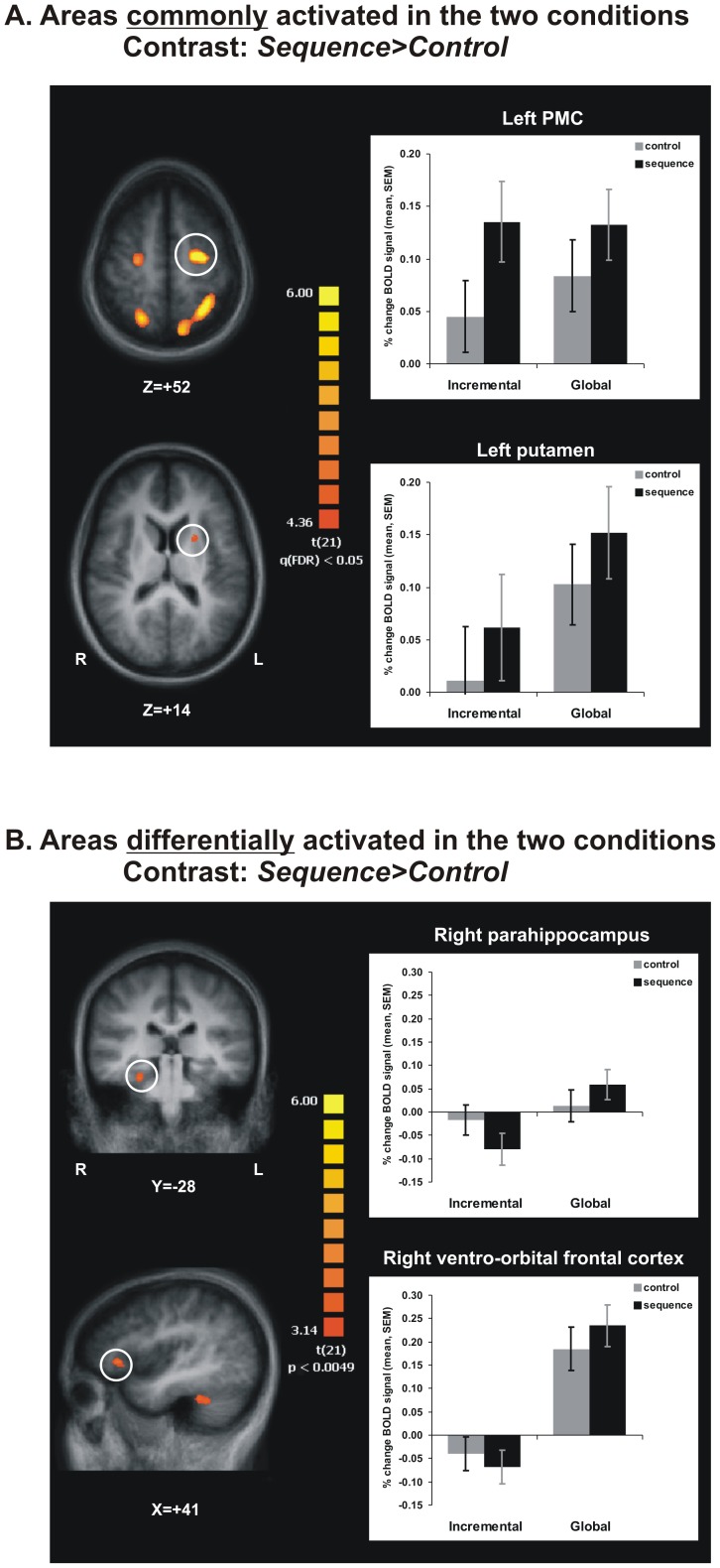
Imaging results. **A**. Areas in the cortico-striatal network activated more during Sequence than Control blocks, and that are commonly found in the two groups. **B**. Areas in ventro-orbital prefrontal cortex and parahippocampus were more activated in participants in the Global than Incremental condition, during Sequence than Control blocks.

While contrasts were computed on the whole brain and the complete pattern of results is reported in Table 2, our description and discussion of the results will focus mostly on the cortico-striatal network and mediotemporal areas. The contrast [Global vs. Incremental], performed for both experimental conditions combined, did not reveal any activation cluster which surpassed the statistical threshold (minimum t-value *t_(21)_* = 4.30; FDR corrected) in either direction (i.e. Global>Incremental or Incremental>Global). The contrast [*Sequence>Control*] performed for both groups together identified 5 clusters of activation (Figure 4A, Table 2), one in the left putamen, and the other four, respectively, in the supplementary motor areas (SMA) and precuneus (Brodmann area BA7) bilaterally. Furthermore, in each of these clusters, the BOLD signal change was significantly higher during the sequence than the control blocks, for each group (main effect of block; all *p<0.05*). In addition, in the left SMA cluster only, there was a significant interaction effect [*F_1,20_ = 6.04; p<0.05*], indicating that the difference between the two types of blocks was higher in the Incremental than in the Global condition.

**Table 2:** Brain regions activated by contrast [*Sequence blocks>Control blocks*].

Contrasts and regions	Talairach coordinates			Volume (mm^3^)	Maximum t
**Sequence>Control blocks (all subjects)**					**t** ***(21)***
Right precuneus (BA7)	21	−62	49	2698	6.12
Right SMA (BA6)	24	−14	53	730	5.29
Left precuneus (BA7)	−26	−55	49	5952	7.00
Left SMA (BA6)	−24	−12	54	2147	7.68
Lentiform nucleus (putamen)	−21	6	16	140	4.87
**Sequence>Control (Global>Incremental)**					**t** ***(21)***
Right STG (BA21)	54	−22	−2	830	3.84
Right culmen (cerebellum)	39	−37	−26	465	4.58
Right VLPFC (BA47)	39	29	1	224	4.45
Right declive (cerebellum)	33	−61	−11	665	4.33
Right parahippocampal gyrus (BA35)	27	−28	−20	347	3.90
Right PCC (BA31)	18	−55	22	342	4.05
Left culmen (cerebellum)	0	−52	−5	532	4.18
Left declive (cerebellum)	−3	−64	−11	153	3.62

SMA – supplementary motor area.

STG – superior temporal gyrus.

VLPFC – ventro-lateral prefrontal cortex.

PCC – posterior cingulate cortex.

The same contrast analysis carried out to assess group differences revealed no region in which subjects in the Incremental group showed more activation than those in the Global group. In 8 clusters, however, the difference in BOLD signal between sequence and control blocks was significantly greater in the Global than the Incremental group (Figure 4B, Table 2). Half of these clusters were found within the cerebellum, with the remaining being located – in ventro-orbito prefrontal (BA47), posterior cingulate cortices (BA31), superior temporal (BA21) and parahippocampal gyri (BA35). Furthermore, the contrast [*Sequence>Control*] performed separately within each group revealed significant bilateral striatal activation in the Global, but not in the Incremental group.

Overall, the results of the block-based contrasts indicate that the participants in both conditions engaged the cortico-striatal network (putamen, SMA, parietal) significantly more during the sequence than the control blocks. In addition, participants exposed to the Global learning strategy during their training engaged significantly more the ventro-orbital prefrontal cortex, cerebellum, as well as the medial and lateral temporal areas. Also, these participants seemed to activate more the striatum bilaterally during sequence than during control blocks.

### Imaging results: event-related contrasts analysis

**Figure 5:**
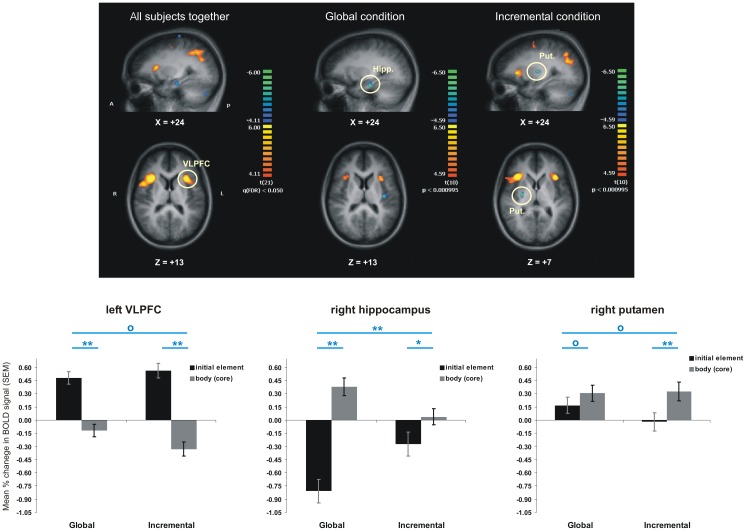
Areas more activated during initial than the subsequent elements of a sequential chunk (orange regions), as well as areas activated more during the core than initial chunk element (blue regions). The bar graphs show the percentage change in blood oxygenation level dependent (BOLD) signal in selected regions, for each group and type of trials. Blue stars indicate significant statistical differences (p<0.05 for one star and p<0.001 for two stars), whereas blue circles indicate no significant difference.

The contrast [*initial SEQ>body SEQ*], between brain activation during the initial element versus that during the core elements of a chunk of the target sequence, revealed that the two groups shared an extensive network of commonly activated regions both when initiating a sequential cluster, as well as when executing the elements forming the core of a chunk (Table 3). On one hand, the common network activated during chunk initiation (the orange-yellow areas in Figure 5, left column) included frontal and prefrontal areas almost exclusively (with the exception of inferior parietal lobule bilaterally). Of these, the activation in the ventrolateral prefrontal clusters (VLPFC – encompassing areas BA44, BA45 and BA47) and anterior insular cortex (INS – BA13), bilaterally, was the most interesting as these brain regions are known to be implicated in a wide variety of language and short-term memory functions that are very much sequential in nature. A detailed analysis of the BOLD signal change in these regions also revealed a significant effect of the type of trial (initial vs. body) [i.e. *F_1,20_ = 44.74*, *p<0.05* in left VLPFC/INS marked in Figure 5, left column], but no significant main effect of group [*F_1,20_ = 1.46*, *p>0.05*], and no significant group * trial type interaction [*F_1,20_ = 0.05*, *p>0.05*]. The trial type effect was significant in both groups [*F_1,10_ = 27.12*, *p<0.05* for Incremental and *F_1,10_ = 19.44*, *p<0.05* for Global]. In addition, we found a significant negative correlation between the difference in BOLD signal in this ROI and the difference in reaction time for the type of trial (initial vs. body), both when we considered the raw reaction time (R^2^ = 0.29, p<0.01), as well as within-group z-score for reaction time (R^2^ = 0.47, p<0.001). On the other hand, the common network involved in executing the chunk's' core (the blue areas in Figure 5, left column) included mostly frontal and temporal areas, both medially and laterally. Among these regions, the results revealed a cluster in the left hippocampus (marked in Figure 5, left and middle columns), where we not only observed the expected significant main effect of trial type [*F_1,20_ = 95.67*, *p<0.05*], but also a significant trial type and group interaction [*F_1,20_ = 30.98*, *p<0.05*]. The latter interaction resulted from a significantly higher difference between activation during the core elements than during the initial element of a chunk among people trained in the Global condition [*F_1,10_ = 130.37*, *p<0.05*] compared to those who trained in the Incremental group [*F_1,10_ = 8.10*, *p<0.05*]. Such effect was observed both when performing the interaction contrast for the entire brain (trial type by condition – Table 4), as well as when analyzing the imaging data separately for each group (Table 5). Importantly, this hippocampal cluster could still be observed in the Global group (Figure 5, middle column), but did not survive the statistical threshold in the Incremental group condition (Figure 5, right column). In fact, with the exception of one cluster located in semilunar lobule in cerebellum, all other clusters observed when pooling the data from both group together, could be found in the Global, but not the Incremental group when performing the same contrast separately for each group.

**Table 3:** Brain regions activated in both groups by contrasts [*Initial element>Core chunk*] and [*Core chunk>Initial element*].

Contrasts and regions	Talairach coordinates			Volume (mm^3^)	Maximum t
**Initial element>Core chunk (all subjects)**					**t** ***(21)***
Right IPL (BA40)	41	−41	38	9173	8.48
Right VLPFC (BA44/BA45/BA47)/INS	40	13	14	6384	7.69
Right DLPFC (BA9)	37	36	33	744	5.72
Right SFG (BA9)	2	6	50	5619	6.72
Left IPL (BA40)	−31	−51	39	6930	7.18
Left SMA (BA6)	−28	−10	49	779	6.50
Left VLPFC (BA44/BA45/BA47)/INS	−33	17	12	3011	7.23
Left PMC (BA6)	−42	−2	29	1002	5.27
Left DLPFC (BA9)	−44	29	37	445	5.00
**Core chunk>Initial element (all subjects)**					**t** ***(21)***
Right MTG (BA21)	61	−11	−8	869	5.58
Right semi-lunar lobule (cerebellum)	31	−69	−35	1007	5.57
Right IFG (BA47)	35	19	−17	368	5.87
Right hippocampus	24	−17	−18	555	6.74
Right medial FG (BA10)	2	52	4	421	4.73
Left medial FG (BA8)	−6	50	40	2091	6.98
Left amygdala	−21	−8	−14	1221	6.20
Left MFG (BA10)	−42	46	−3	859	5.08
Left STG (BA38)	−45	16	−22	1791	7.76
Left angular gyrus (BA39)	−46	−68	35	1041	5.55
Left MTG (BA21)	−58	−5	−10	1944	6.15

IPL – inferior parietal lobule.

VLPFC – ventro-lateral prefrontal cortex.

INS – anterior insular cortex.

DLPFC – dorso-lateral prefrontal cortex.

SFG – superior frontal gyrus.

SMA – supplementary motor area.

PMC – premotor cortex.

MTG – middle temporal gyrus.

IFG – inferior frontal gyrus.

Medial FG – medial frontal gyrus.

STG – superior temporal gyrus.

MFG – middle frontal gyrus.

**Table 4:** Brain regions activated by contrast [*Core chunk>Initial element*] more in Global than in Incremental group.

Contrasts and regions	Talairach coordinates			Volume (mm^3^)	Maximum t
**Core chunk>Init. elem. (Global>Increm.)**					**t** ***(21)***
Right STG (BA22)	57	−7	2	402	4.57
Right posterior insular cortex (BA13)	39	−17	16	702	4.09
Right IFG (BA47)	31	16	−17	201	5.17
Right PCC (BA31)	12	−56	21	3030	6.67
Right hippocampus	25	−20	−19	113	4.74
Right medial FG (BA10)	21	52	12	181	3.57
Left precuneus (BA31)	−12	−50	35	703	4.07
Left SMA (BA6)	−26	10	66	269	4.60
Left PMC (BA6)	−31	−12	68	716	4.78
Left posterior insular cortex (BA13)	−36	−20	13	740	5.08
Left MTG (BA39)	−48	−64	27	4179	5.20
Left postcentral gyrus (BA2)	−49	−27	56	710	4.91
Left postcentral gyrus (BA43)	−53	−7	14	133	3.75
Left STG (BA22)	−60	−23	3	154	3.69
Left MTG (BA21)	−62	−3	−15	438	4.89

STG – superior temporal gyrus.

IFG – inferior frontal gyrus.

PCC – posterior cingulate cortex.

Medial FG – medial frontal gyrus.

SMA – supplementary motor area.

PMC – premotor cortex.

MTG – middle temporal gyrus.

**Table 5:** Brain regions activated by contrast [*Core chunk>Initial element*] separately within each group.

Contrasts and regions	Talairach coordinates			Volume (mm^3^)	Maximum t
**Core chunk>Initial element (Incremental)**					**t** ***(10)***
Right MFG (BA11)	36	34	−11	177	6.17
Right lentiform nucleus (putamen)	26	−12	7	119	8.13
Right pyramis (cerebellum)	25	−73	−34	122	5.69
Left STG (BA38)	−45	19	−24	108	6.92
**Core chunk>Initial element (Global)**					**t** ***(10)***
Right MTG (BA21)	59	−9	−6	1245	9.27
Right posterior insular cortex (BA13)	37	−19	16	305	6.56
Right IFG (BA47)	32	17	−18	396	9.22
Right hippocampus	24	−20	−18	521	10.18
Right SMA (BA6)	21	−14	72	122	6.52
Right SPL (BA7)	11	−52	71	231	5.62
Left medial FG (BA8)	−8	48	43	1211	9.73
Left medial FG (BA9)	−4	59	26	280	6.78
Left precuneus (BA31)	−15	−46	33	321	6.63
Left amygdala	−19	−8	−18	382	6.44
Left SMA (BA6)	−29	−15	70	302	8.75
Left posterior insular cortex (BA13)	−38	−17	15	441	6.82
Left MTG (BA21)	−49	4	−20	3376	9.25
Left angular gyrus (BA39)	−47	−67	30	4327	12.88
Left IFG(BA10)	−45	44	−5	180	5.37
Left STG (BA38)	−44	15	−35	135	6.00
Left postcentral gyrus (BA2)	−47	−28	58	147	5.82

MFG – middle frontal gyrus.

STG – superior temporal gyrus.

MTG – middle temporal gyrus.

IFG – inferior frontal gyrus.

SMA – supplementary motor area.

SPL – superior parietal lobule.

Medial FG – medial frontal gyrus.

Previous experiments and the current models of motor sequence posit that the striatum is involved in this process, but these claims are usually based on block-related contrasts. In our experiment we did not detect any activations in the striatum in response to the event-related contrasts, performed either when comparing the two groups or pooling their data together. However, when performing the contrast [*initial SEQ>body SEQ*] for subjects in the Incremental group only, we observed, as expected, a cluster in posterior right putamen. Detailed analysis in this region revealed that even though the interaction between experimental conditions and trial type was not significant [*F_1,20_ = 0.30*, *p>0.05*], the individuals in the Incremental group showed significantly more activation in this region when putting sequence elements into well integrated chunks than when executing the movements preceding these chunks [*F_1,10_ = 17.18*, *p<0.05*]. Participants in the Global condition, however, did not show such a significant difference between trial types [*F_1,10_ = 0.37*, *p<0.05*]. Nevertheless, in Global condition, the changes in BOLD signal for both types of events in striatum (i.e. initial element and cluster core) were positive and significantly above baseline, indicating that the participants in this condition recruited the striatum to integrate together the initial and the core elements of the cluster.

In conclusion, we observed that the two groups shared a common neuronal network during chunk initiation, located almost exclusively in frontal and prefrontal regions, and another one during the execution of the chunk core, located mainly in parieto-temporal lobes. Regarding the dissociation between the roles of striatum and hippocampus in chunking, our results suggest that the hippocampus contributes to the execution of chunks, but not to their initiation, and that such an effect was observed to a greater extent when people had extensive practice with the same sequence (Global condition), then when they were exposed to it gradually (Incremental condition). The activity in posterior putamen was significantly higher than baseline for both the first and the remaining elements of a chunk in the Global group (indicating the integration of the first chunk element with the others), and only during the execution of the chunk core for subjects in the Incremental group.

## Discussion

We manipulated the training regimen of two groups of participants in order to test the roles of cortico-striatal circuits and MTL in chunking of sequence elements during the late phase of motor learning. Although both groups learned the same motor sequence by the end of training, they differed in terms of their reaction time and variability in motor responses. Individuals in the Global condition, who developed a stable sequence representation, were faster and less variable than their counterparts in the Incremental condition. During the last four runs of the imaging session, which took place the day after the 4 days of training were complete, the two groups were similar in terms of motor performance. The only observed difference between the two groups being observed at the level of motor sequence reproducibility, which was, as expected, higher in the Global than in the Incremental condition. When performing contrasts taking into account the whole block of motor performance, we noted that the two groups activated significantly more the cortico-striatal areas during the execution of the target sequence than during the control condition. However, performing the active sequence in the Global group resulted in greater putaminal activity, bilaterally.

The event-related analysis allowed us to dissociate between the cerebral activity specific to the initiation of sequence chunks compared to that related to execution of the chunk cores. Both groups engaged, to the same extent, a prefrontal network when initiating sequential chunks, with a large volume of activation seen in the ventrolateral prefrontal cortex, bilaterally. In contrast, the neural correlates specific to the execution of well integrated chunks were localized predominantly in the MTL, including hippocampus and amygdala; in most of these regions, the activation was greater in the Global than in the Incremental condition.

Behavioral results indicate that despite having different training regimens and different ways of learning the same sequence, both groups reached a similar level of performance during the imaging session with some differences in the variability of performance. Changes in reaction time during learning of the sequence and its variability within a single training session were used in the past as indicators of learning or movement execution strategies employed by the subjects [7], [38], [40]. The scope of having different behavioral training regimens was, in our study, to foster a stable, albeit complex representation of the motor sequence in the Global group and to gradually build up such a representation to develop in the Incremental group. Both measures of reaction time and variability were lower in the Global than in the Incremental group in the 8^th^ training session, hence suggesting that the latter participants had a more stable representation of the sequence, as well as a stable execution strategy. Interestingly, chunk's length was significantly smaller in the Global than the Incremental group, suggesting that a stable and complex representation of the sequence can rest on small clusters, similar to knowing by heart a long phone number, which is divided in smaller parts.

During the imaging session, the mean reaction times were comparable in the two groups; the only difference being observed only at the level of reaction time variability. This indicates that, at this stage, subjects in the two groups were able to execute the sequence at the same speed. Yet participants in the Global group displayed less variability, hence a more stable performance, suggesting that they developed a different strategy to perform the task [38].

Another view that may explain the difference between the two groups that arose from the specificity of behavioral training could relate to the extent of consolidation of motor memory. Previous studies have shown that periods of diurnal or nocturnal sleep can contribute to the consolidation of motor sequences, expressed as spontaneous gains between sessions in the absence of practice, stability of the performance or resistance to interference [6], [11]–[14], [41]. Given that subjects in the Global group were exposed to the same sequence during their behavioral training, it is likely that they benefited to a greater extent from memory consolidation induced by time and night sleeping periods; those in the Incremental group could only benefit from the consolidation due to the night of sleep between the last behavioral and the subsequent imaging sessions. Thus, differences in stability of the motor performance (i.e. variability) may also reflect differences in the motor skill consolidation during sleep and in the amount of practice on the same sequence.

The imaging results from the block-related analysis are in accord with previous findings showing that the putamen is involved in the self-generation of multiple novel actions especially when no clear distinction is available between competitive alternatives [42], [43] (i.e. in our case, each individual response was performed with the same frequency throughout the task; thus each response had the same probability of occurring). Furthermore, dorsal striatum activity increases have been reported for category judgments in the context of greater category uncertainty [44]. In the context of the present study, the nature of the training regimen for the Incremental group makes it so that the earlier movements of the sequence are more heavily biased (practiced) than the latter ones, while they carry an equal value in the Global group, possibly explaining the greater recruitment of putamen in the latter group.

The greater recruitment of the striatum and its cortical projections in both group is concordant with the neurobiological model of motor sequence learning proposed by Doyon and collaborators [2], [5], [45], which predicts an increased cortico-striatal activity in the later phases of motor sequence learning. The present results show that the plastic changes in this specific network are relatively independent of the subject's learning strategy, given that they were present in both groups; this finding is novel in the motor sequence learning literature. In contrast, the Global, but not the Incremental learning strategy, led to recruitment of additional areas in the ventro-lateral prefrontal cortex, cerebellum and temporal lobe. This additional ventro-lateral prefrontal activation seems to parallel findings in the literature on executive processes, which propose that the fronto-striatal loops are implicated in processing feedback information requiring a change in strategy [46], in planning and rule shifting when no clear clues are provided by the environment [43], and in switching from a planned response to a novel [46] or a more difficult one [47]. The greater involvement of temporal and cerebellar areas in Global than in Incremental learning group is consistent with recent findings showing that synchronization of motor performance (i.e. higher temporal reproducibility of the sequence) correlates positively with the level of cerebellar activity in later stages of motor learning [19] and that inter-regional connectivity tends to increase with practice after weeks of training, despite the fact that the regional cerebral activity may decrease over time [48]. Thus, the recruitment of additional areas outside the cortico-striatal network seems to support the development of a stable long-term representation and execution strategy of the motor sequence.

The novelty of our findings relies, however, in the event-related analysis, which permitted us to dissociate between the neural substrate mediating chunk initiation *versus* chunk execution. As stated before, the first element in a chunk has always a slower reaction time relative to the subsequent chunk elements due to the fact that transitions between clusters reflects a starting cost or higher memory load as a previous cluster is discarded and another one is loaded [20], [36], [37]. Here, we showed that despite differences in variability or in execution strategies between the two experimental conditions, subjects in both groups recruited the ventrolateral PFC/anterior insula, bilaterally, when executing the first element of a sequential chunk. In the neuroimaging literature on motor learning to date, there are only a few studies employing an event-related analysis on sequence chunking [34] and the findings support the recruitment of frontoparietal networks in chunk initiation or segmentation. In contrast, the literature on set/task-shifting [46], [49], [50] and on rule/goal selection [51]–[53] has more such studies and they identified the VLPFC as one of the regions involved in rule shifting/selection processes. Although conjectural, it is possible that the latter activation in this area is related to an executive process related to chunk management, independently of the sequence representation. The fact that the areas identified in chunk initiation also included anterior insula is in line with recent findings which suggest that this part of the insular cortex has an important role in high-level cognitive and attentional processes [54]. In this context, it is hypothesized that anterior insula detects salient events and acts as a hub between large scale networks in order to facilitate proper allocation of attention and working memory resources. Consistent with this role, there was a significant negative correlation between the difference in BOLD signal and the difference in reaction time between the initial element and the body of the chunk, suggesting that the better or smoother the chunking process, the higher the difference in activity in this region.

A clear dissociation between the two experimental conditions was also observed in regard to the neural substrate associated with the chunking itself, where differences were observed in: hippocampus and other MTL areas in the Global group and in posterior putamen in Incremental condition. Similarly, the sensorimotor putamen was shown elsewhere to be significantly involved in chunk concatenation [34], consistent with findings showing that chunking is heavily dependent on dopaminergic circuits in PD patients [35]. Unlike Wymbs and colleagues [34], who did not manipulate learning strategy, we did not find chunking-related activation in putamen when we combined the two experimental conditions, but only in the Incremental condition, which is a clear indication that it depends on the training regimen. The absence of striatal finding for subjects in the Global group may seem at odds with the neurobiological model of motor sequence learning [2], [5], [45], which predicts increased striatal activity in the later stages of learning. However, following the argument described above regarding *differential* striatal activation in both groups, we propose that since all movements are equally trained in the global group, and that the chunks are indeed small, it is equally difficult to distinguish the chunk body from the element initiating the chunk, and therefore striatal requirement is similar for both conditions. On the other hand, it is possible that the first trial of a chunk is significantly differentiated from the body of the chunk in the incremental group, which leads to more putaminal activity when choosing the next movement of the sequence within the chunk. Therefore, the fact that we did not observe a *differential* activation in striatum for the Global group, when comparing the chunk initiation with the chunk body itself; does not mean that the striatum is not implicated in later stages of learning. In fact, a detailed analysis of the activity in the posterior putamen, which was found to be more activated during the execution of core elements than during the initiation of a chunk in Incremental condition, showed that the activity level in the same region was above baseline (rest period) during both types of events for the participants in the Global group (Figure 5). Thus, this finding does support and even expand the role of striatum in the later stages of learning, by showing the involvement of this structure in clustering or chunking of sequence elements in both experimental conditions. In contrast, the pattern of activation in hippocampus was specific for the subjects in Global condition; only in this case, the activity was higher than baseline during chunking indicating that the development of a stable sequence representation is accompanied by a specific change in hippocampal activity. In the past, the recruitment of hippocampus during motor learning tasks was associated with the formation of higher-order associations [9], with the behavioral performance changes as learning progresses [6]– and with overnight, but not over day gains in performance [6], [17]. Our results go one step further: we report here that the activity in the hippocampus is specifically associated with chunking of sequence elements, but only when a stable sequence representation and execution strategy is in place. Of course, given the fact that our scanning session was administered at the end of 4 days of training, it is certain that the learning process in the Global group may have benefited from the consolidation due to four nights of sleep. However, this does not discount the fact that hippocampus was more active only during the execution of the elements that were already well clustered and was actually ‘deactivated’ during the initial chunk elements, thus supporting our claim that its role is in chunking the elements once the sequence representation is stable.

Finally, our results have two major implications for the motor sequence learning literature. First, from a methodological viewpoint, our data suggest that employing an event-related design organized around chunking or clustering of sequence elements may provide insights on the functional roles of various cerebral regions that would not be otherwise revealed by classical block-based contrasts. Second, our findings could be relevant from a clinical point of view concerning the procedural learning capacity of amnesic patients with damage to the hippocampus. It has been shown in the literature that these individuals are capable of learning implicit motor skills [56], [57], including motor sequences. Yet, people with amnesia have also been found to have a deficit in implicitly learning high-order associative information, similar to the second-order conditional probability which exists between elements of the sequence in the current study [58]. Our results indicate that in procedural learning of explicit sequences hippocampus is involved in the development of a stable sequence representation and execution strategy, which may be the basis for actually developing the motor expertise. The explicit memory demands in our task arguably pose a problem for people with anterograde amnesia. Still, there is past evidence that these individuals could perform well an explicit motor sequence task with no visual guidance, provided that the order of the elements in the sequence is determined by a well-known rule [59]. In this case, our data suggests that the use of an incremental learning strategy may be beneficial for these patients.
